# Photoperiodic Modulation in Immune and Reproductive Systems in Japanese Quails (*Coturnix japonica*): A Morphometric Perspective

**DOI:** 10.3390/vetsci9050248

**Published:** 2022-05-23

**Authors:** Khizar Hayat, Ali Raza, Aitzaz Anas, Anas Sarwar Qureshi, Sarmad Rehan, Ameer Hamza Rabbani, Hafiz Faseeh ur Rehman, Abdul Ghaffar Qamar, Tayyab Rehman, Farah Deeba, Amber Salman

**Affiliations:** 1 Jockey Club College of Veterinary Medicine and Life Sciences, City University of Hong Kong, Hong Kong 999077, China; 2 Department of Anatomy, University of Agriculture, Faisalabad 38000, Pakistan; anas-sarwar@uaf.edu.pk (A.S.Q.); drsarmadpk@gmail.com (S.R.); miantyb30@gmail.com (T.R.); 3 Department of Anatomy and Histology, University of Veterinary and Animal Sciences, Lahore 54000, Pakistan; faseeh.rehman@uvas.edu.pk; 4 Department of Clinical Medicine and Surgery, University of Agriculture, Faisalabad 38000, Pakistan; a.raza@uq.edu.au (A.R.); farrah.deeba@uaf.edu.pk (F.D.); 5 House Office, Independent Medical College, Faisalabad 38000, Pakistan; aitzazanas@gmail.com; 6 Department of Surgery, Cholistan University of Veterinary and Animal Sciences, Bahawalpur 63100, Pakistan; ameerhamzarabbani@cuvas.edu.pk; 7 Department of Clinical Sciences, Riphah College of Veterinary Sciences, Lahore 54000, Pakistan; agqamar2424@gmail.com; 8 University Medical and Dental College, The University of Faisalabad, Faisalabad 38000, Pakistan; dr.ambersalman2@gmail.com

**Keywords:** histomorphometry, photoperiod, avian immune system, avian reproductive system, androgens

## Abstract

The present study was designed to elucidate a relationship between lymphoid organs and reproductive activity in male Japanese quails (*Coturnix japonica*) bred in a temperate region of Pakistan (30.3753° N, 69.3451° E) in response to photoperiodic changes. The research focused primarily on the relative morphological changes in primary (thymus and bursa of Fabricius) and secondary (spleen) lymphoid organs with respect to seasonal variations in the histomorphometry of testicular tissue. For this purpose, a comparable number of clinically healthy Japanese quails were exsanguinated during active (April–May), regressive (September–October) and inactive (January–February) reproductive phases. Following an extensive gross measurement of lymphoid and reproductive organs, a histomorphometric analysis was performed on sampled tissues by employing ImageJ^®^ software. Blood was collected for hormonal and leukocytic analysis. One-way ANOVA was used for statistical comparison. Testes had the highest parenchymal development in the active phase (80.66 ± 21.22 µm) and the lowest in the inactive phase (27.80 ± 7.22 µm). Conversely, a percentage change was evident in the sizes of primary (bursa: 61.5%, thymus: 46.9%) and secondary (spleen: 23.9%) lymphoid organs during inactive and active reproductive phases. This study demonstrated that a physiological trade-off is imperative between immune and reproductive systems for optimum survivability and reproductive performance.

## 1. Introduction

The immune and reproductive systems are two major body systems that require extensive resource allocation therefore, trade-offs must be made between them during different physiological states. This phenomenon is particularly evident in seasonal breeders, such as birds [1]. Previous studies have demonstrated that androgens are primarily responsible for immune and reproductive changes in birds subsequent to neuroendocrine stimulation [2]. However, it is still unclear whether this phenomenon is limited to males only or whether estrogen could also play an important role in immune regression during a phase of higher reproductive activity [3,4].

Primary immune organs have been a focus of investigators as an integral part of the trade-off between immune and reproductive systems [5,6]. Despite being the major secondary immune organ, the spleen has been ignored altogether over the years. The spleen’s role is particularly important in birds because it acts as a pure lymphoid organ owing to a greater proportion of white pulp (containing aggregated lymphoid tissue) than red pulp [7,8]. Conversely, in mammals, red pulp comprises a greater portion of the spleen considering its auxiliary function in erythrocyte storage [9,10]. A comprehensive study was required to investigate if the spleen followed a similar trend as primary lymphoid organs. Most studies in this regard have focused on the physiological manifestations attributed to a compromised immune status. The authors in the current study employed the susceptibility toward parasitic infestation as an indirect indication of reduced immunogenic capabilities. It is widely accepted that blood and intestinal parasites could prove to be reasonable indicators of avian immune status. An increased level of parasitic infestations has been observed in birds during their active breeding season [11,12,13]. An anatomical basis of this physiological variation was analyzed among wild birds in a prior study [5]. However, limited literature has been published whereby both gross and histomorphometric parameters were studied concomitantly. Moreover, minimal information has been made available pertaining to the role of hypertrophied parenchyma relative to the organ stroma during active reproductive seasons.

Birds indigenous to tropical regions are known to undergo annual photoperiodic changes causing a reduced level of testosterone in plasma. This physiological change enhances immunity and depresses overall reproductive functioning during reproductively inactive season [14,15]. It has been reported that melatonin can enhance immune status of birds but at this time, the extent of this increase in melatonin countering the testosterone-mediated decrease in immunity during active reproductive season is not well-understood [16]. There has been some discussion on the number of reproductive phases in an annual breeding season [17]. Keeping in mind relatively recent studies, birds are thought to undergo two major transitions in one cycle with three distinct reproductive states, which are active, regressive and inactive [6]. The Japanese quail was selected for this study as a model bird because of its important role as an essential poultry bird for meat and egg production evolved over the last few decades [18,19].

Most prior studies relating to variations in the immune status of birds in relation to reproductive phases were conducted on wild birds [16,20,21,22]. Recent studies report a negative impact of stress on immune status causing its fairly mild regression [23,24]. Therefore, a study focused on investigating stress-associated immune compromises in commercially farmed birds is imperative to confirm the aforementioned trend in domesticated birds. The current study aimed to determine a seasonal relationship between immune and reproductive statuses of Japanese quails. To remove the impact of environmental factors and influences associated with breeding seasonality, captive birds were inducted in the present study. It was hypothesized that the immune and reproductive systems make a trade-off at a morphometric level during the annual breeding cycle. To observe this change in immune status, morphometric parameters of primary and secondary lymphoid organs were investigated in relation to a variation in testicular morphology, as a proxy for breeding seasonality in domestic birds. To support our observations, hematological and hormonal analyses were performed to correlate the morphometric changes of immune and reproductive organs with immune cells in blood and plasma testosterone levels.

## 2. Materials and Methods

### 2.1. Sample Collection

This study was conducted at the Department of Anatomy, University of Agriculture Faisalabad, Pakistan after approval from the Institutional Biosafety and Bioethics Committee (IBC, DGS No. 4141-44). Commercially available adult male birds (*n* = 50) were decapitated in each reproductive phase, namely active breeding season (Spring; April–May), regressive season (Autumn; September–October) and inactive season (Winter; January–February). To minimize the age variation among adult birds, feather analysis (primary and secondary coverts) was performed along with obtaining complete history (date of purchase of quail chicks) from the farmer [25]. After purchase, birds were transferred to the cages in animal house for 7 days for acclimatization. During this period, optimum environmental conditions such as light (5 Lux, 15.5 h per day), humidity (65%), temperature (25–27 °C) and ventilation were provided to minimize stress [26,27,28]. Feed and water were offered to the birds *ad libitum*. After acclimatization period, birds were decapitated, and organs were removed from the carcass via conventional postmortem technique. During slaughtering, blood sampling was also performed for lymphocytic and hormonal analysis. Organs were first washed in normal saline and then preserved in fixatives. Bursa of Fabricius, thymus and spleen were preserved in 10% aqueous solution of buffered formalin (10% NBF), while testes were preserved in Bouin’s solution for effective and rapid fixation [29]. Organs were kept in the fixatives for at least 72 h until further processing.

### 2.2. Hematological Analysis

Blood was collected in two separate vacutainers containing ethylenediaminetetraacetic acid (EDTA) and coagulation gel (Bolton^®^, MIDAS, Karachi, Pakistan). Lymphocyte percentage was counted after making a thin smear of uncoagulated blood under the microscope. EDTA-mixed blood was used for white blood cell enumeration with Natt–Herrick (N&H) method for total leukocyte count [30]. Blood was mixed with N&H solution in a micropipette and then poured in Neubar’s hemocytometer for analysis under the microscope at 400X magnification.

### 2.3. Gross Morphometry and Histological Sample Preparation

Gross morphometric values were measured before putting the organs in preservative fluids to avoid any variation from the in vivo state. Weight of the organs was measured via digital weighing balance, while length, width and diameter were measured using vernier calipers (VCs). The following formula was used to measure the relative weight of the organs in relation to the total weight of individual:

Relative Weight (RW) of organ: 100/Weight of bird × Absolute Weight (AW) of organ.

Zero error of VCs was removed prior to measuring the organs. After gross morphometric analysis, organs were immersed in fixative for 72 h. Conventional paraffin embedding technique was used for preparation of microscopic slides. Organs were dehydrated via increasing gradients of alcohol from 70% to 100%. Clearing of this alcohol was performed first using a mixture of xylene and alcohol (xylol) and later with 100% xylene. Paraffin infiltration was performed after melting of paraffin in hot air oven. Finally, paraffin embedding was conducted, and blocks were left overnight for further processing. Tissues were sectioned on manual rotary microtome (Microm HM315^®^, Rankin Biomed, Holly, MI, USA) at 4–6 µm thickness. After mounting of tissues on microscopic slides, a hotplate was used to dewax the tissue. Routine staining was performed using Hematoxylin and Eosin solution to stain the tissues on microscopic slides, and coverslips were mounted using DPX^®^ (Sigma-Aldrich, Karachi, Pakistan) [29].

### 2.4. Histomorphometric Analysis

Compound light microscope (Anti-Mould SE^®^, Nikon, Tokyo, Japan) with camera was used to obtain the images for histomorphometric analysis of samples, which was performed manually using computerized software ImageJ (Research Services Branch, National Institute of Mental Health, Bethesda, MD, USA) [31]. Software was calibrated using a microscopic image of the stage micrometer. Multiple measurements were taken per slide to minimize human error. All measurements were saved, and mean values were calculated for graphical representations.

### 2.5. Hormonal Analysis

Serum was harvested from blood in gel vacutainers and stored at −20 °C for further analysis. Radioimmunoassay (RIA) was used for the quantification of serum testosterone levels using a commercially available kit (AccuBind ELISA Microwells Testosterone Kit^®^, Monobind, Lake Forest, CA, USA). Briefly, 10 µL of serum samples were taken in designated well in which 50 µL of testosterone enzyme reagent containing 0.7 mL of testosterone in steroid conjugate buffer. After 30 s, 50 µL of testosterone biotin solution was added to those wells, and the microplate was incubated at 27 °C for one hour. After decanting the microplate and drying with absorbent paper, it was washed with 350 µL of washing buffer three times. In each well, 100 µL of 3,3′,5,5′-tetramethylbenzidine substrate (TMB substrate) was added, and the microplate was incubated at 27 °C for 15 min. After incubation, 50 µL of Stop Solution was mixed, and the plate was shaken for 15–20 s. Finally, the microplate was read at 450 nm with reference filter set at 630 nm wavelength using a digital microplate reader (SHE3000^®^, Perfemed, Chemux Bioscience Inc., South San Francisco, CA, USA).

### 2.6. Statistical Analysis

Data were statistically analyzed using SPSS Version 21^®^ (IBM, Armonk, NY, USA). One-way analysis of variance (ANOVA) was used with post hoc Tukey’s test for the analysis. Confidence interval (CI) was maintained at 95% (*p* < 0.05).

## 3. Results

### 3.1. Anatomical Parameters

The absolute mass of the testes was significantly highest in the active reproductive phase (1.28 ± 0.16 g) as compared to the regressive (0.46 ± 0.12 g) and inactive phases (0.44 ± 0.14 g) with the latter ones differing insignificantly from each other. Interestingly, the relative weight of the testes differed significantly during all three seasons (1.74 ± 0.17%, 1.2 ± 0.12% and 0.55 ± 0.13%, respectively). Similar results were observed for the length and width of the testes where all three phases demonstrated significant changes from each other (Table 1).

**Figure 1:**
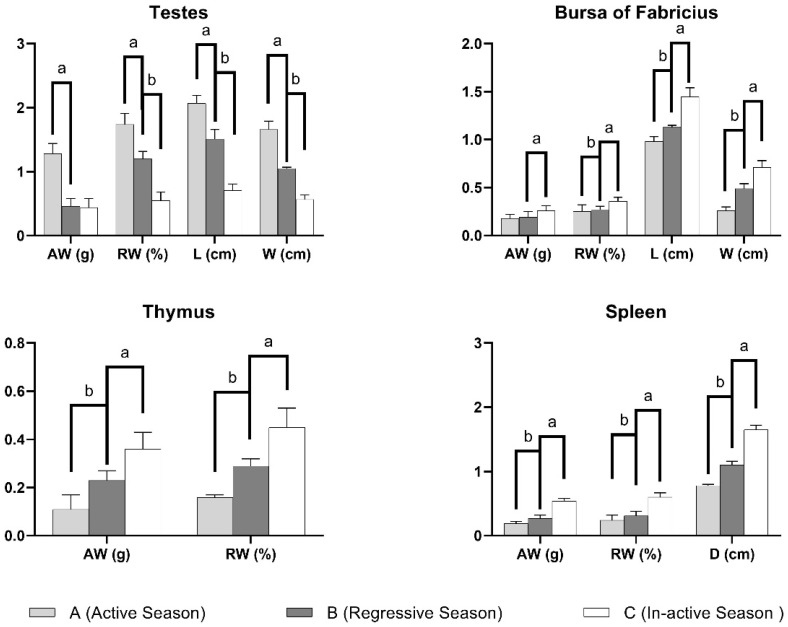
Graphical representation of gross morphometric values of organs (testes, bursa of Fabricius, thymus and spleen) in different phases of a reproductive season. AW: Average weight, RW: Relative weight, L: Length, W: Width and D: Diameter. All values are represented as means ± SEM, and parameters with different superscripts ^(a,b,c)^ are significantly different from each other (*p* < 0.05).

Contrarily, the absolute mass of the bursa of Fabricius was highest in the inactive breeding phase (0.26 ± 0.05 g), while it was statistically indifferent during the regressive (0.19 ± 0.06 g) and active breeding seasons (0.18 ± 0.04 g). The relative weight was highest in the inactive breeding season (0.36 ± 0.04%) and differed significantly during all three seasons. The length and width of the bursa followed a similar trend as that of the absolute weight and relative weight, respectively (Figure 1).

The absolute weight and relative weight of the thymus followed an identical pattern where inactive, regressive and active reproductive phases differed significantly from each other (Table 1).

The spleen showed a similar trend where the absolute weight was statistically different during all three reproductive phases, i.e., active, regressive and inactive (Figure 1). The relative weight was highest in the inactive breeding phase (0.60 ± 0.07%); however, it differed insignificantly during the regressive and active seasons. The diameter of the spleen was significantly different during all three seasons (Table 1).

### 3.2. Histological Parameters

**Figure 2:**
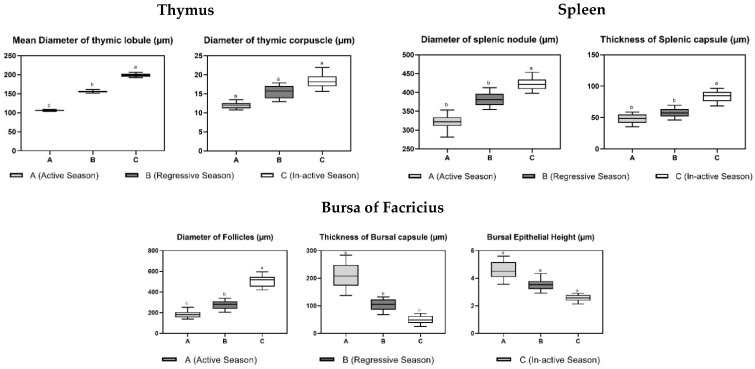

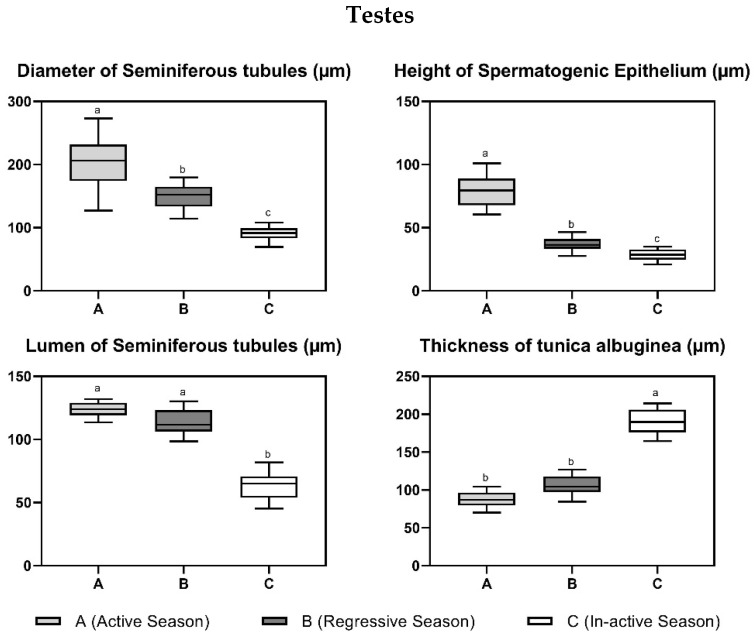
Boxplot representing histomorphometric parameters in immune organs during active (**A**), regressive (**B**) and inactive breeding phases (**C**). All values are represented with their median, and upper and lower quartiles are shown in the above figure. Overlapping whiskers between different seasons show a relatively lower level of significance as compared to the ones in the which the difference is greater. Parameters with different superscripts ^(a,b,c)^ are significantly different from each other (*p* < 0.05).

**Figure 3:**
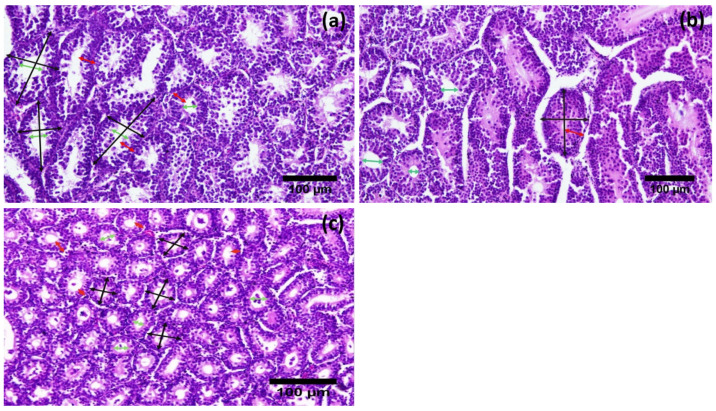
Photomicrographs of testes of Japanese quails during different breeding phases of annual reproductive cycle. (**a**) Active breeding phase, (**b**) Regressive breeding phase and (**c**) Inactive breeding phase. Curved black arrows demonstrate the measurement of diameter; green arrows show the lumen of seminiferous tubules, and red arrows show height of spermatogenic epithelium. (H&E 200X).

**Figure 4:**
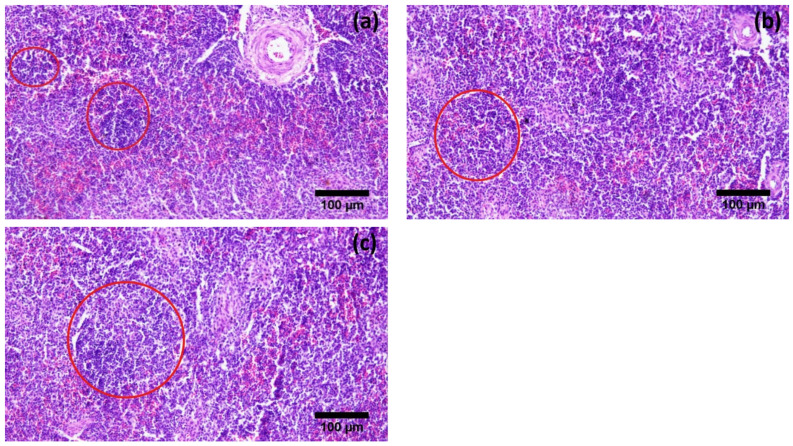
Photomicrographs of spleen of Japanese quails during different breeding phases of annual reproductive cycle. (**a**) Active breeding phase, (**b**) Regressive breeding phase and (**c**) Inactive breeding phase. Red circles show the splenic nodule (present in white pulp) of spleen (H&E 200X).

**Figure 5:**
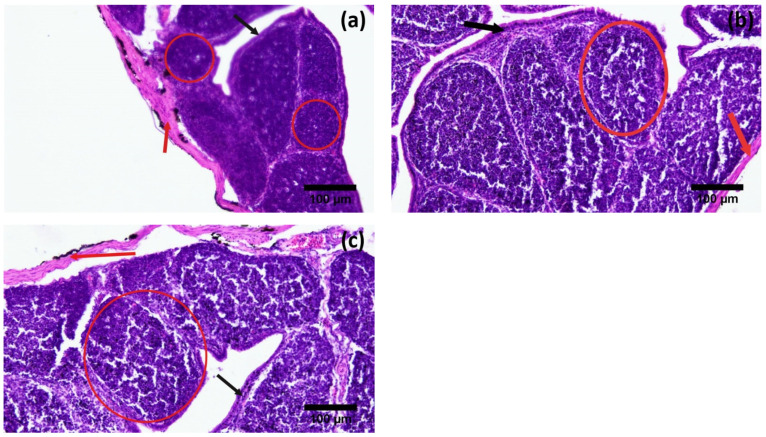
Photomicrographs of bursa of Fabricius of Japanese quails during different breeding phases of annual reproductive cycle. (**a**) Active breeding phase, (**b**) Regressive breeding phase and (**c**) Inactive breeding phase. Red circles show the bursal nodule; black arrows show the epithelium of bursa, and red arrows show bursal capsule (H&E 200X).

Histological examination of the testes showed that the diameter of seminiferous tubules (203.37 ± 65.22 µm) and the height of the spermatogenic epithelium (80.66 ± 21.22 µm) were significantly greater during the active reproductive season as compared to the regressive and inactive seasons. On the other hand, the luminal diameter of the seminiferous tubules was significantly different in the regressive season (114.4 ± 15.93 µm) as compared to the nonactive season (63.24 ± 19.11 µm). The difference was insignificant between regressive and active breeding phase (114.4 ± 15.93 µm and 122.71 ± 9.98 µm, respectively) (Figure 2). Contrarily, the thickness of the tunica albuginea was significantly higher in the inactive reproductive phase (191.10 ± 26.85 µm) as compared to the regressive and active phases (Table 2, Figure 3). A comparative analysis of histomorphometric parameters of the spleen demonstrated an opposite trend to that of the testes. The diameter of the splenic nodule (426.52 ± 29.47 µm) and thickness of capsule (82.19 ± 14.57 µm) were significantly higher in the nonactive reproductive phase, while they remained statistically non-significantly different during the regressive and active seasons (Figure 4). The histological analysis of the bursa showed that the diameter of the follicle and thickness of the capsule were significantly different during the three reproductive seasons. The diameter of the bursa follicle was highest in the nonactive reproductive season (508.52 ± 13.06 µm), while it was the lowest in the active reproductive season (195.5 ± 19.54 µm). Contrarily, the diameter of the bursa capsule was found to be highest in the active reproductive season (213.46 ± 23.49 µm), while it was the lowest in nonactive season (48.51 ± 7.94 µm). The height of the epithelium showed no significant difference (Figure 5).

The diameter of the thymic lobule differed significantly during the three reproductive seasons (*p* ≤ 0.05). The highest values were observed during the inactive reproductive phase (199.01 ± 7.03 µm), while the lowest value was observed during the active reproductive phase (105.63 ± 2.59 µm) with the regressive season (156.73 ± 5.47 µm) showing a gradual change in the diameter of the thymic lobule. In contrast, the difference in diameter of the thymic corpuscle was insignificantly different during all three reproductive seasons (Table 2).

### 3.3. Hematological Parameters

Immunological parameters such as Total Leukocyte Count (TLC) and Lymphocyte Count (LC) were significantly different during the three reproductive phases with the highest number being in the inactive breeding phase (189.3 ± 6.53 k/µL, 137.18 ± 4.92 k/µL) and the lowest number in the active breeding phase (Table 3).

### 3.4. Hormonal Analysis

The testosterone level was detected as being significantly higher during the active reproductive phase (2.45 ± 0.035 ng/mL) with a gradual reduction in the regressive phase, and the lowest values were seen in the nonactive reproductive phase (Table 3).

## 4. Discussion

Our study revealed that birds experienced the highest level of morphological development with regards to their reproductive organs during the active breeding phase. Similar annual changes have been reported in *Coturnix japonica*, *Perdicula asiatica* and *Numidia meleagris* [7,9,18]. The decrease in weight of the testes was significant as the season changed from active to regressive. However, the difference was relatively less during the inactive season. Conversely, the length and width of the testes decreased at an almost comparable rate during both physiological transitions. This variation in rate of decrease in the morphometric parameters of the testes was interesting to observe in terms of the functionality of the organ. Increased gonadal activity during the active reproductive season ensures that there would be maximum production of active spermatozoa, which can be used for a higher frequency of copulation [32]. The authors postulate that this variation in percent decline of weight during the two transitions was due to the presence of active spermatozoa in the seminiferous tubules during the active phase, which contributes toward the weight of the organ. An analysis of the photomicrographs shows that spermatozoa reach their minimum number during the regressive stage and disappear completely in the inactive season. After the regressive season, the number of spermatozoa in the seminiferous tubules decrease rapidly as the testicular size shrinks. The interstitial tissue has been shown to continue regressing at almost the same level which in turn causes a further decrease in the length and width of the organ [33,34].

This hypothesis was further tested and proven in the histomorphometric analysis of testicular tissue in which the height of the germinal epithelium (GE) was highest during the active breeding phase as opposed to the regressive and inactive ones. However, the decrease in the height of the spermatogenic epithelium (SE) was almost double when moving from the active to the regressive phase as compared to moving from the regressive to inactive phase (53% and 27%, respectively). This supports our initial hypothesis that due to meiotic activity, spermatogenic hyperplasia occurs in the testes, which adds to the weight of the organ exponentially. An increase in testicular parenchyma is directly related to an increase in the weight of the organ. These results were eerily similar to the ones reported in other avian species [5,35]. Moreover, we also observed that the diameter of the seminiferous tubules (STs), in keeping with the overall trend, differed slightly where the percent decrease in the first transition was relatively less than the second one, i.e., 25% and 39%, respectively. Due to the accumulation of free spermatozoa in the lumen of the STs and the hyperplasia of spermatogenic cells, the basement membrane of the STs may be stretched and even after the active season, it does not return to its normal stage immediately [36]. This leads to a relative slower decrease in the diameter of the STs as compared to the height of GE. The diameter of the SE lumen and thickness of the tunica albuginea followed the exact opposite trend where the active season showed the minimal values compared to the other two. It is understandable that due to an increase in the epithelial height, the lumen was decreased; however, as Figure 2 shows, the rate of increase in the luminal diameter was far greater during the first transition as compared to the second one. This corroborates the previously discussed phenomenon where a delay in regression/return of the basement membrane to its normal state was observed [33]. This theory needs further testing; therefore, the authors suggest an investigation employing special stains to differentiate between the types of connective tissue present in the basement membrane and their relative changes observed during different phases of the breeding season.

Morphometric parameters of the lymphoid organs showed a completely opposite trend, whereby the highest values were observed during the inactive breeding phase. The relative increase in the weight of the spleen and bursa was higher in the second transition as compared to the first one. Contrarily, an increase in the size of the thymus was relatively greater in the first seasonal change as compared to the second one. Similar trends were observed with other morphometric parameters of immune organs such as the length and width of the bursa and the diameter of spleen where the inactive breeding state showed the highest values as compared to the other two. These results agree with those observed in different species, such as willow tits [37]. Comparable results were reported for the splenic parameters of Indian tropical bush quails (*Perdicula asiatica*) as well [16]. In birds, the seasonal regression of the thymus is relatively more pronounced as compared to the bursa of Fabricius. During the active breeding season, birds can afford to reduce the thymic lymphoid tissue even beyond acceptable thresholds in certain dire situations. Consequently, an abrupt change in thymic weight is not in itself a measure of its activity, rather it is an organ’s attempt to return to its normal size after a significant decrease during the active breeding phase [38].

An organized immune system is present in specialized immune organs such as the bursa of Fabricius (bursal follicle), thymus (thymic lobule) and spleen (splenic nodule). Only one study has been reported regarding the histomorphometry of the thymus during different breeding seasons in any adult avian species [38]. Overall, the lymphoid tissue followed a similar trend in which the highest values were observed during the inactive season, and the lowest ones were in the active season. These results corroborated findings of a prior study conducted in *Numidia meleagris* [5,6]. However, unlike our present observations, the percent increase in their morphometric values varied significantly for all three organs. Both the thymic lobule and splenic nodule exhibited similar behavior with a steep decline in size (diameter) during the transition from the active to the regressive state (32% and 15%, respectively). However, a relatively lower rate was observed during the transition from the regressive to the inactive state (21% and 10%, respectively). This may correlate with the resource allocation from the reproductive system to the immune system. Immediately after the active breeding season, birds need to boost their immune system to cope with the climatic stress and increase their survivability for the next active season. To achieve this, a rapid reallocation of resources occurs, and thus we see a sharp increase in the lymphoid content. As the body moves from the regressive to the inactive season, other external factors, such as nestling stress, start cutting into the energy consumption thus causing the rate of increase in lymphatic aggregates to plumate [39]. Contradictory results were observed in the case of the bursa of Fabricius where a higher percentage change in bursal diameter was observed during the second transition as compared to the first one (46% and 28%, respectively). It may be related to the fact that lymphocyte production keeps on increasing in the bursa as it is the major primary lymphoid organ even in the inactive season to maintain the lymphocyte number in the body for higher chances of survivability. The same phenomenon can be observed in migratory birds where a significant decrease in the size of the spleen is observed during the migratory season [40]. However, this theory needs to be tested through a comparative analysis of the lymphocyte–lymphoblast population in the bursal follicles during all three phases. The capsule of the lymphoid organs is made up of connective tissue and is considered a stromal component as compared to the lymphoid aggregates, which are parenchymal in nature. The capsule thickness also varied in the three reproductive phases; however, this change was not identical to that of the parenchymal change during the same period, which leads us to believe that both vary independently from each other during the same period. Moreover, the change in the parenchymal components was much higher and more pronounced as compared to the stromal components, which supports our initial theory that it is the parenchymal content that shows major plasticity during different reproductive phases [41].

It was also interesting to compare the morphometric changes in primary and secondary lymphoid organs. As adult birds were used for this study, we were expecting to have a greater degree of morphometric variation (especially parenchymal changes) in the spleen as compared to the thymus and bursa. The reason behind this was that primary lymphoid organs tend to have a greater degree of functionality during the early years of life as compared to later ones [42]. However, this study revealed that the degree of variation was much higher in the case of primary lymphatic organs as compared to secondary ones. This is consistent with previous findings in which a variation was observed in the regression of primary immune organs in different avian species with some showing more regression compared to others [43]. The bursal and thymic lymphatic lobules doubled their diameter from the active season to inactive season, while the change in the splenic lobule was only around 75%. Contrarily, we also observed that the decrease in the lymphoid content of the thymus and bursa was much more pronounced during the active reproductive phase. We think this might be due to the difference in the types of cells present in these organs [44]. However, this needs further investigation where advanced techniques such as immunohistochemistry (IHC) can be used for the differentiation of these cells.

One of the factors that researchers explored while trying to analyze the immune status of wild birds is the extent of parasitic infestations (intestinal and blood infections) [11,12]. Previous studies have shown that wild birds face a higher level of parasitemia during the active breeding phase [6,16]. This conforms to the hypothesis that not only the lymphoid organs, but also the peripheral status of immunity undergoes major regression during higher reproductive activity. Similar results were seen in our study where the lymphocyte percentage and TLC were lowest in the active breeding season. Increased levels of leukocytes including lymphocytes during the inactive reproductive phase suggest that not only the initial responders of the immune response (neutrophils) but also the artillery (lymphocytes) is enhanced. These higher levels are required for proper bloodborne immune activity [45].

The results in this study confirm previous reports suggesting that the testosterone level changes with climatic conditions due to activation or regression of the gonads during the sexually active and inactive seasons, respectively [16,45,46]. The results reveal that the serum testosterone level was significantly higher during the active reproductive season as compared to the nonactive reproductive season. This further corroborated the initial hypothesis that an increase in testosterone was directly related to the lower levels of immune parameters.

Immunohistochemical (IHC) techniques could be employed in future endeavors to observe the differences in the ratios of different immune cells and the variation in the number of lymphocytes during active breeding seasons. There are some reports that state that this phenomenon is not readily observable in female birds. Therefore, future studies could also involve female birds to investigate the role of estrogen in controlling the morphometric values of immune organs.

## 5. Conclusions

The study revealed that Japanese quails follow a breeding cycle with varying degrees of reproductive activity such as active, regressive and inactive reproductive phases. This is evident both from the morphometric parameters and from the hormonal analysis. This change in reproduction is directly related to the immune status of birds. A trade-off between the reproductive status and immune statuses of birds must be made due to finite energy reserves in the body where an increase in activity of one system leads to a decrease in another and vice versa. Moreover, it was also observed that primary and secondary lymphoid organs do not show the same level of morphometric changes during this seasonal variation. A variation in morphometry was much more pronounced in primary lymphoid organs as compared to secondary ones. In addition, this variation is not limited to the morphometric parameters of organs only. It was also observed that the number of circulating immune cells (blood-borne) also follow a similar trend with the highest number being in the nonactive reproductive phase and vice versa.

## Figures and Tables

**Table 1:** Gross anatomical measurements of different organs of Japanese quails (*Coturnix japonica*) during different reproductive phases.

Parameter	Annual Reproductive Season		
	Active Season (April–May)	Regressive Season (September–October)	Nonactive Season (January–February)
Testes			
Absolute Weight (g)	1.28 ± 0.16 ^a^	0.46 ± 0.12 ^b^	0.44 ± 0.14 ^b^
Relative Weight (%)	1.74 ± 0.17 ^a^	1.2 ± 0.12 ^b^	0.55 ± 0.13 ^c^
Length (cm)	2.07 ± 0.12 ^a^	1.51 ± 0.15 ^b^	0.71 ± 0.1 ^c^
Width (cm)	1.66 ± 0.13 ^a^	1.05 ± 0.02 ^b^	0.57 ± 0.07 ^c^
Bursa of Fabricius			
Absolute Weight (g)	0.18 ± 0.04 ^b^	0.19 ± 0.06 ^b^	0.26 ± 0.05 ^a^
Relative Weight (%)	0.25 ± 0.07 ^c^	0.27 ± 0.035 ^b^	0.36 ± 0.04 ^a^
Length (cm)	0.98 ± 0.05 ^b^	1.13 ± 0.02 ^b^	1.45 ± 0.09 ^a^
Width (cm)	0.26 ± 0.04 ^c^	0.49 ± 0.05 ^b^	0.71 ± 0.07 ^a^
Thymus			
Absolute Weight (g)	0.11 ± 0.06 ^c^	0.23 ± 0.04 ^b^	0.36 ± 0.07 ^a^
Relative Weight (%)	0.16 ± 0.01 ^c^	0.29 ± 0.03 ^b^	0.45 ± 0.08 ^a^
Spleen			
Absolute Weight (g)	0.19 ± 0.03 ^c^	0.27 ± 0.05 ^b^	0.54 ± 0.04 ^a^
Relative Weight (%)	0.24 ± 0.08 ^b^	0.31 ± 0.07 ^b^	0.60 ± 0.07 ^a^
Diameter (cm)	0.78 ± 0.02 ^c^	1.1 ± 0.06 ^b^	1.65 ± 0.07 ^a^

All values are represented as mean ± SD, whereby different superscripts ^(a,b,c)^ are used to identify a statistical significance of *p* < 0.05.

**Table 2:** Histomorphometric analysis of Japanese quails (*Coturnix japonica*) during different reproductive phases.

Parameter	Annual Reproductive Season		
	Active Phase (April–May)	Regressive Phase (September–October)	Nonactive Phase (January–February)
Testes			
Diameter of seminiferous tubules (µm)	203.37 ± 65.22 ^a^	151.71 ± 28.22 ^b^	91.04 ± 11.41 ^b^
Height of spermatogenic epithelium (µm)	80.66 ± 21.22 ^a^	37.06 ± 9.67 ^b^	27.80 ± 7.22 ^c^
Lumen of seminiferous tubules (µm)	122.71 ± 9.98 ^b^	114.4 ± 15.93 ^b^	63.24 ± 19.11 ^a^
Thickness of tunica albuginea (µm)	86.81 ± 17.75 ^b^	105.29 ± 22.15 ^b^	191.10 ± 26.85 ^a^
Bursa of Fabricius			
Diameter of follicles (µm)	195.5 ± 58.54 ^c^	271.13 ± 69.39 ^b^	508.52 ± 91.06 ^a^
Thickness of capsule (µm)	213.46 ± 78.49 ^a^	101.42 ± 37.08 ^b^	48.51 ± 23.94 ^c^
Epithelial height (µm)	4.57 ± 1.09 ^a^	3.61 ± 0.73 ^a^	2.53 ± 0.4 ^a^
Thymus			
Mean diameter of thymic lobule (µm)	105.63 ± 2.59 ^c^	156.73 ± 5.47 ^b^	199.01 ± 7.03 ^a^
Diameter of thymic corpuscle (µm)	12.11 ± 1.34 ^a^	15.16 ± 2.71 ^a^	18.79 ± 3.25 ^a^
Spleen			
Diameter of splenic nodule (µm)	324.58 ± 33.34 ^b^	383.74 ± 28.86 ^b^	426.52 ± 29.47 ^a^
Thickness of capsule (µm)	46.68 ± 11.96 ^b^	57.74 ± 12.03 ^b^	82.19 ± 14.57 ^a^

All values are represented as mean ± SD, whereby different superscripts (^a,b,c^) are used to identify a statistical significance of *p* < 0.05.

**Table 3:** Immune and hormonal analysis of Japanese quails (*Coturnix japonica*) during different reproductive phases.

Parameter	Annual Reproductive Season		
	Active Phase (April–May)	Regressive Phase (September–October)	Nonactive Phase (January–February)
Immunological Analysis			
Total Leukocyte Count (×10^3^/µL)	109.5 ± 1.95 ^a^	157.25 ± 2.37 ^b^	189.3 ± 6.53 ^c^
Lymphocyte Count (×10^3^/µL)	79.34 ± 2.79 ^a^	113.94 ± 3.16 ^b^	137.18 ± 4.92 ^c^
Hormonal Analysis			
Serum Testosterone (ng/mL)	2.45 ± 0.035 ^a^	0.60 ± 0.024 ^b^	0.27 ± 0.011 ^c^

All values are represented as mean ± SD, whereby different superscripts ^(a,b,c)^ are used to identify a statistical significance of *p* < 0.05.

## Data Availability

The data generated in this study is already added in the tables of this article. If you need any further information, please feel free to contact authors.
